# Epigenetic deregulation of BORIS and CTCF in breast cancer

**DOI:** 10.1186/1756-8935-6-S1-P1

**Published:** 2013-03-18

**Authors:** Iliana Alcalá Moreno, Ernesto Soto-Reyes, Daniela Morales-Espinosa, Rodrigo González Barrios, Hétor Aquiles Maldonado-Martinez, David Cantú de León, Lissania Guerra-Calderas, Clementina Castro, Luis A Herrera

**Affiliations:** 1 Unidad de Investigación Biomédica en Cancer - Instituto Nacional de Cancerología - Instituto de Investigaciones Biomédicas, Universidad Nacional Autónoma de México; D. F., México

## Background

CCCTC binding factor, also known as CTCF, has been described as a gene transcription promoting factor. On a genome–scale this protein is able to mediate long-range chromatin interactions enabling the regulation of the expression of domain genes. On a gene-scale CTCF is associated with an insulation function that counter propagation of methylation and repressive histone marks, specially in promoters associated to CpG islands of genes such as *BRCA1*, *ER*, *Rb*, *p16*, *p53*, *and miR-125b1*. For this reason CTCF dissociation is associated to epigenetic silencing. *CTCF* has a paralog gene, *CTCFL* or BORIS (*CTCF-like* or Brother of the Regulator of Imprinted Sites), whose over-expression has been reported in neoplastic tissue such as breast cancer. BORIS is endogenously expressed only in testes, and its gene is epigenetically silenced in all other cells via DNA methylation. While CTCF is distributed throughout the nucleus, BORIS is localized in both nucleolus and nucleus.

## Materials and methods

The main interest of our work was to determine whether BORIS and CTCF were deregulated in breast cancer. First we analyzed the expression of both genes in three different cell lines (MCF7, MDA-MB-231, MCF10A) by RT-PCR. In order to evaluate the sub-cellular localization of BORIS and CTCF we performed immunofluorescent detection of both proteins in the three cell lines. As a way of establishing a correlation between cell lines and cancer/neoplastic tissue, we performed an immunohistochemical staining of BORIS and CTCF in tissue samples from breast cancer patients. Sodium bisulfite assays were carried out in order to elucidate whether the changes in DNA methylation were responsible for the over-expression of BORIS.

## Results

The results suggest that BORIS is abnormally over-expressed in cancer cell lines. Data showed that in the non-neoplastic cell line BORIS was found within the nucleolus, whereas in MCF7 it was only found in the nucleoplasm. CTCF localization showed no difference between cell lines. In the breast cancer samples BORIS was found in the cytoplasm while CTCF was not found in the aggressive cancer tissue. These data point out that there might be a deregulation both at protein localization and gene expression levels. We evidenced a loss of methylation in the BORIS promoter from the samples of breast cancer patients in contrast with the normal tissue samples. A reactivation of BORIS expression in the samples that exhibited a loss of DNA methylation.

## Conclusions

BORIS and CTCF may be deregulated in a model of breast cancer.

